# Posterior deltoid-sparing approach for displaced inferior or posterior glenoid fossa fractures: technical note and case series

**DOI:** 10.1038/s41598-024-56974-7

**Published:** 2024-03-18

**Authors:** Eic Ju Lim, Ho-Seung Jeong, Kook-Jong Kim

**Affiliations:** Department of Orthopaedic Surgery, Chungbuk National University Hospital & College of Medicine, 776, 1sunhwan-ro, Seowon-gu, Cheongju-si, Chungcheongbuk-do Republic of Korea

**Keywords:** Glenoid, Scapula, Fracture fixation, Deltoid muscle, Organ sparing treatments, Patient positioning, Anatomy, Medical research

## Abstract

Scapular surgery has usually been performed through the posterior Judet approach. This approach allows access to the entire posterior scapular body, but causes significant soft tissue damage and detaches the deltoid muscle. To date, there has been no clinical study of a deltoid-preserving approach to access the joint for displaced postero-inferior glenoid fractures (Ideberg type II or Ib). We describe an easy and less invasive approach to the postero-inferior glenoid fossa.

## Introduction

Thus, the purpose of this study was to introduce an easy and less invasive approach to the postero-inferior glenoid fossa and to evaluate the clinical and radiological outcomes following open reduction and internal fixation (ORIF) of displaced glenoid fossa fractures using this posterior deltoid-sparing approach.

### Surgical technique

**Figure 1:**
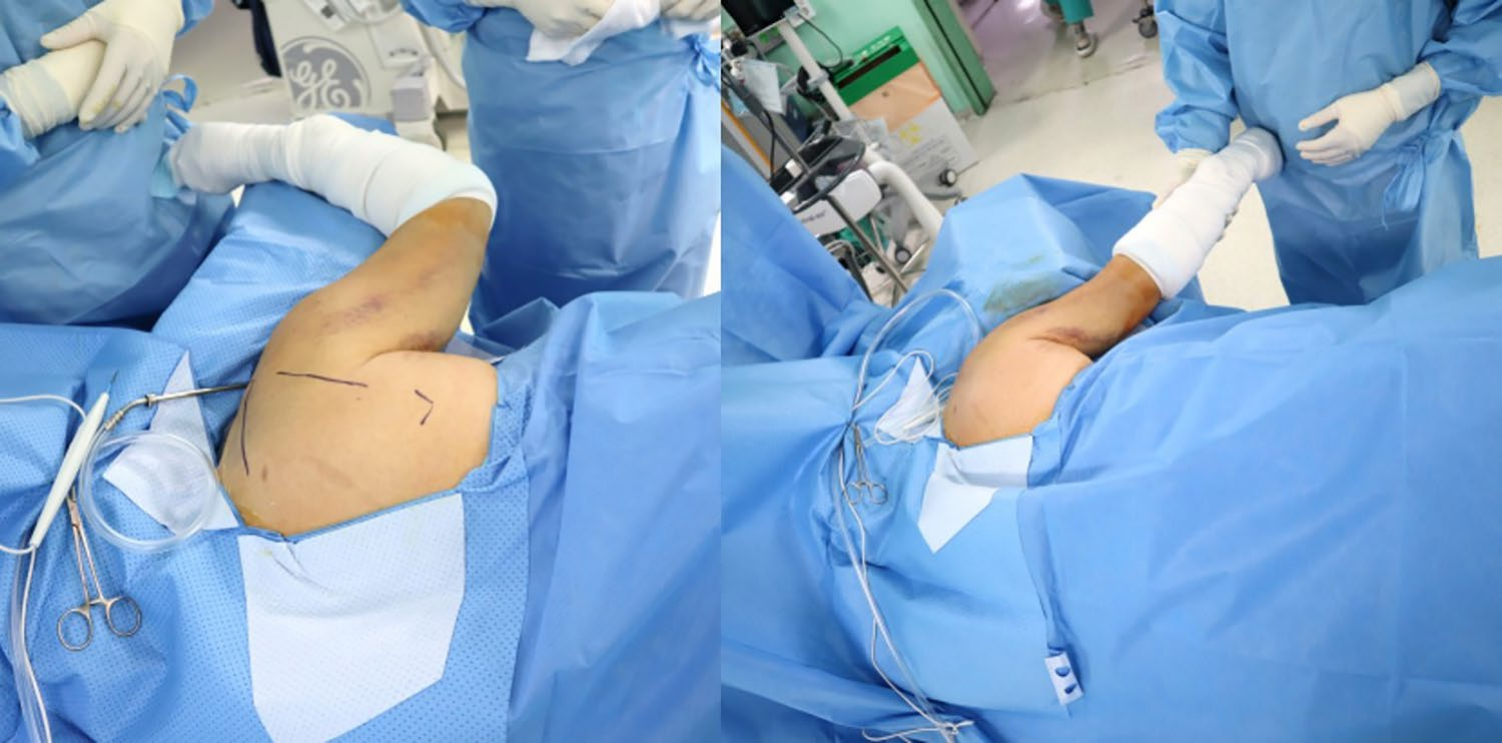
The patient is positioned in the prone position with a pad under the chest, and with a higher pad applied on the fracture side than on the intact side. We adjusted the height of the pad or tilted the table so that scapular plane is parallel to the floor.

**Figure 2:**
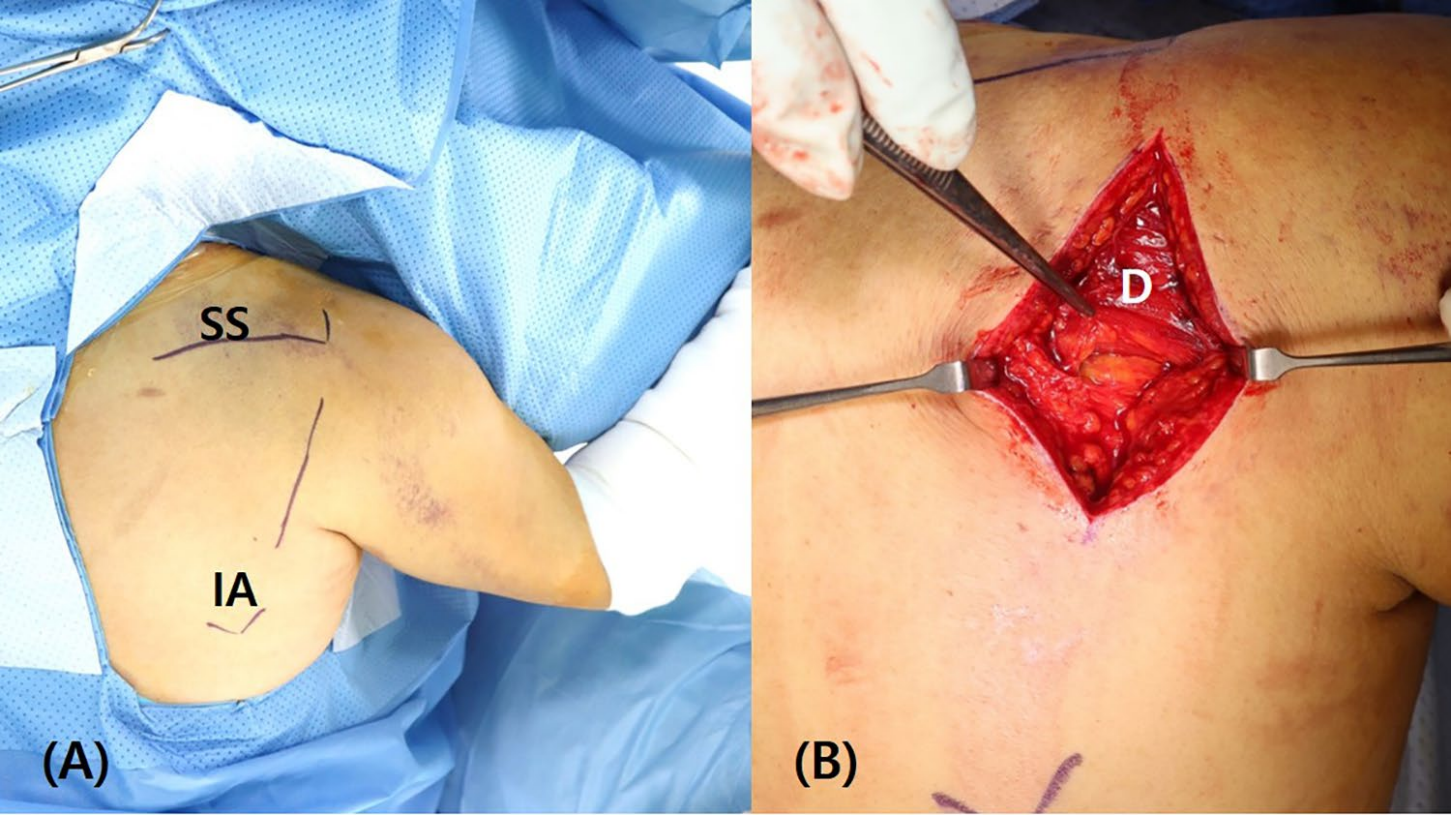
(**A**) An 8‒10-cm straight skin incision is made over the lateral border of the scapula, with the arm in 90-degree abduction. (**B**) Dissection of deltoid fascia is performed and the inferior border of the deltoid muscle is visualized. SS: scapular spine, IA: inferior angle of scapula, D: deltoid.

Patients were placed in the prone position with the pad under the chest, with a higher pad applied on the fracture side than on the intact side (Fig. 1). We adjusted the height of the pad or tilted the table so that the scapular plane was parallel to the floor, and so that C-arm fluoroscopy could easily access the glenoid from the contralateral side or cephalic side. The arm was abducted and draped free. An 8‒10-cm straight skin incision was made over the lateral border of the scapula, with 90-degree abduction of the arm (Fig. 2), as this incision can facilitate expansion of the incision to the scapular body and inferior angle.

**Figure 3:**
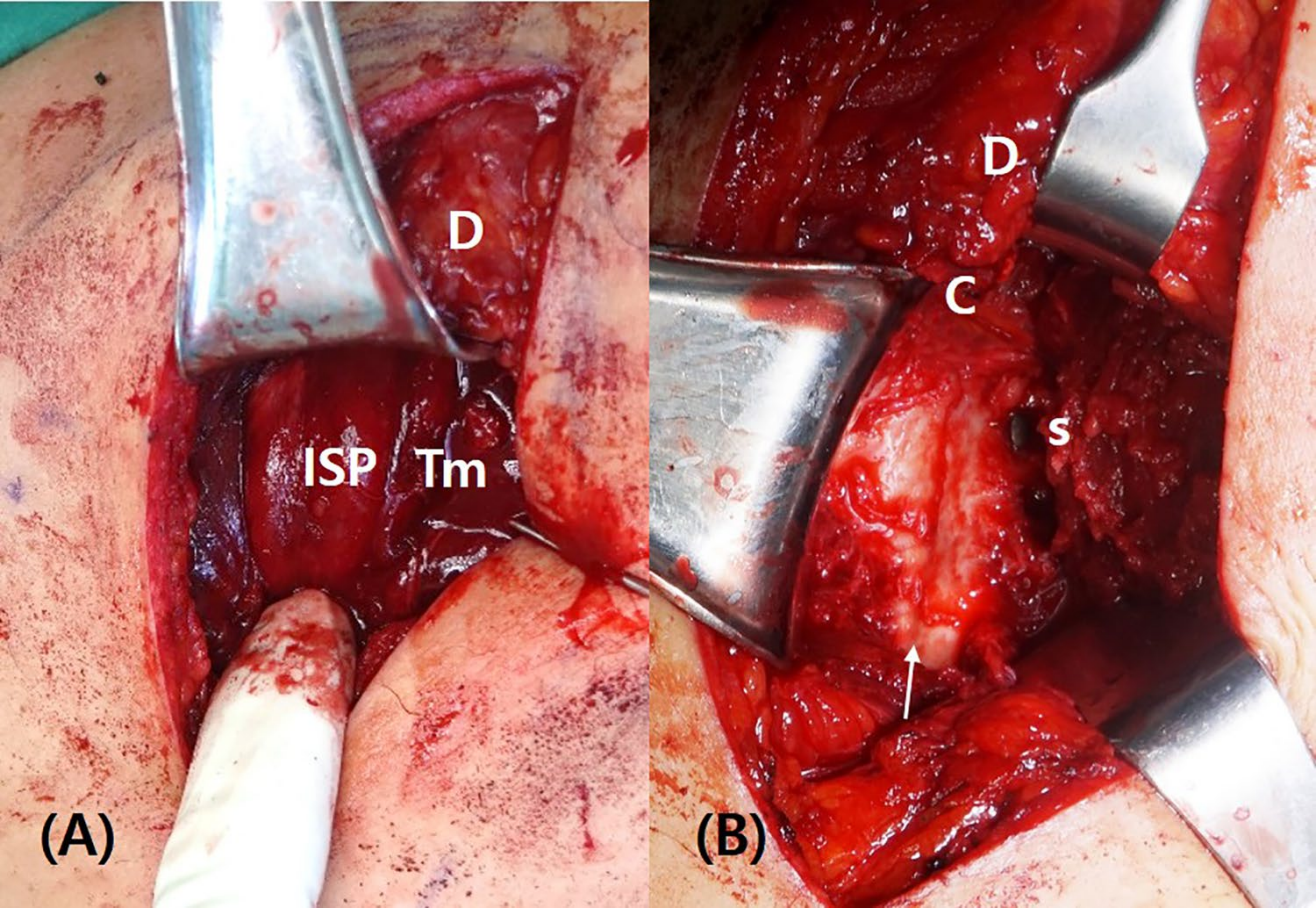
(**A**) The deltoid muscle is retracted cephalad, and the underlying infraspinatus and teres minor muscles can be identified. (**B**) The infraspinatus muscle is elevated proximally, and the teres minor muscle is retracted distally to expose the lateral part of the infraspinous fossa and posterior joint capsule. After the fracture was reduced, it was fixed with a screw at the 6 o'clock position. D: deltoid, ISP: infraspinatus, Tm: teres minor, C: repaired posterior capsule, s: inserted screw, arrow: reduced fracture line.

The deltoid fascia was dissected and the inferior border of the deltoid muscle was visualized (Fig. 2). The deltoid muscle was retracted cephalad, and the underlying infraspinatus and teres minor were identified. Dissection was performed between the infraspinatus and teres minor muscles, and the infraspinatus muscle was elevated proximally, while the teres minor muscle was retracted distally to expose the lateral part of the infraspinous fossa and posterior joint capsule (Fig. 3). To obtain confirmation of the joint surface, the inside of the joint was assessed by applying a vertical incision to the joint capsule. The glenoid can be observed by inserting Kolbel glenoid retractor between the incised capsules and pushing the humeral head laterally. The incision in the capsule should not be made below the infraglenoid tubercle to prevent damage to the axillary nerve. When the incision was extended for wider access to the inferior scapular body, the circumflex scapular artery could be identified on the lateral border of scapular, and was tied to prevent bleeding.

**Figure 4:**
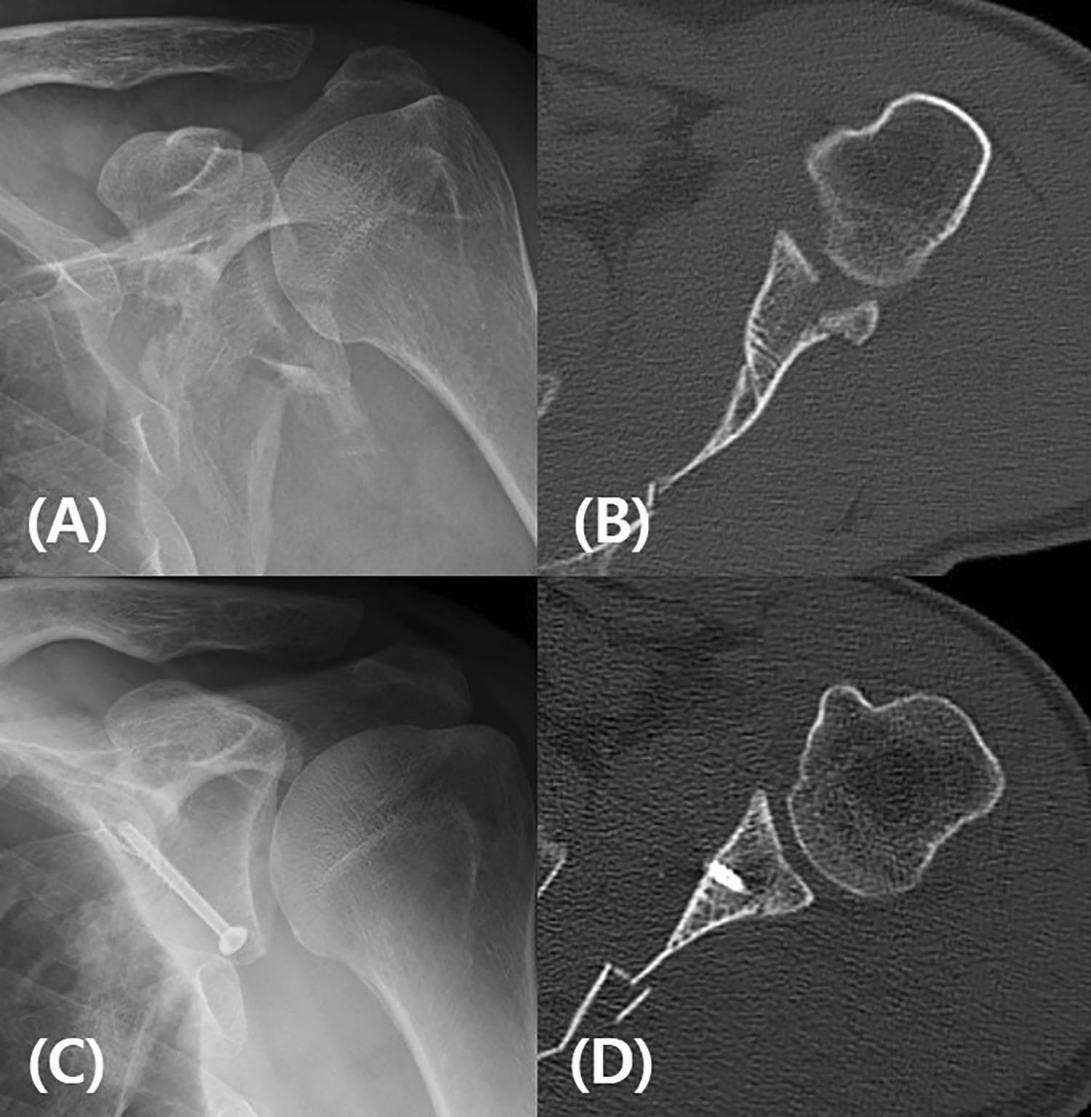
(**A**) Preoperative simple radiography and (**B**) three-dimensional computed tomography (3D CT) show a displaced postero-inferior glenoid fossa fracture. (**C**) A 4.0-mm partial-thread cannulated screws are inserted, and (**D**) postoperative CT shows excellent congruency of the articular surface of glenoid fossa.

The fracture was reduced under direct vision of both the intra-articular and extra-articular aspect of the fracture. The fragments were held temporary in the reduced position with 1.6-mm Kirschner wire (K-wire). One or two 4.0-mm partial-thread cannulated screws were inserted through the guide pin or temporary K-wire after reaming (Fig. 4). When a screw or pin was inserted at the 6 o’clock position of the glenoid, the long head of triceps must be retracted laterally to protect the axillary nerve. After the fixation, closure of capsule was performed by simple interrupted sutures. If complete closure was difficult due to severe capsular injury around the fracture, only partial repair was performed.

### Postoperative rehabilitation

All patients followed the same rehabilitation regimen. The shoulders were immobilized for 6 weeks with an abduction brace. Range of motion (ROM) exercises for the elbow, wrist, and fingers were started immediately after surgery. Passive ROM exercises began at 1 week after surgery, within the patient’s tolerance of pain. Active-assisted ROM exercises were allowed after the patients were weaned off the brace. Muscle strengthening was started at 12 weeks.

### Case series

This retrospective study was approved by the institutional review board of our hospital (IRB no. CBNUH 2020-11-009-002) and the need for patient informed consent was waived due to its retrospective design. All research was performed in accordance with relevant guidelines and local regulations.

Between January 2019 and December 2020, sixty patients presented with a scapular fracture to our hospital. Twenty patients had posterior or inferior glenoid fractures (Ideberg type II or Ib). Fifteen of these patients underwent ORIF with the posterior deltoid-sparing approach. Postoperative computed tomography (CT) scans were performed for evaluation of the reduction state. Ten patients were followed up for more than 1 year; these included three female and seven male patients. The mean age of the patients was 59.8 years (range: 27‒85). The mean follow-up was 14.1 months (range: 12‒34 months) (Table 1).

**Table 1:** Patient data.

Patient number	Age (years)	Sex	Injured side	BMI	Cause of injury	Associated injury
1	60	Male	Right	28.57	Falling from a height	Cerebral hemorrhage, rib fractures, thoracic spine fractures, clavicle shaft fracture
2	27	Female	Left	23.92	Falling down	
3	31	Male	Left	30.39	Bicycle accident	
4	83	Male	Left	21.22	Bicycle accident	Cerebral contusion, rib fractures, tibio-fibular fracture
5	44	Male	Left	23.86	Falling down	Acromion fracture
6	55	Male	Left	33.37	Falling from a height	Skull fracture, cerebral hemorrhage, cervical spine fracture
7	79	Male	Right	22.36	Pedestrian traffic accident	Cerebral contusion, femur shaft fracture
8	56	Male	Left	24.56	Falling from a height	Skull fracture, cerebral hemorrhage, rib fractures, clavicle shaft fracture
9	85	Female	Right	19.31	Falling down	Shoulder posterior dislocation
10	78	Female	Right	27.47	Falling down	

*BMI* body mass index (kg/m^2^).

Six patients had additional associated injuries, mostly of the thorax and the brain. Four patients had severe traumatic injury (injury severity score > 15)^5^. All patients had two-part articular fractures. The mean preoperative fracture gap and step-off were 9.1 ± 4.1 mm (range, 4.4‒15.2 mm) and 4.7 ± 2.8 mm (range, 1.5‒8.8 mm), respectively. Associated ipsilateral clavicle fractures had occurred in two patients, and acromial fracture had occurred in one patient. Three patients had multiple rib fractures, and two patients had spine fractures. One patient had an axillary nerve injury and recovered at 3 months postoperatively. The other injuries are listed in Table 1. The surgical treatment was conducted on an average at 5.6 days (range, 1–16 days) after trauma.

The mean postoperative‒preoperative fracture gap and step-off were 0.8 ± 0.5 mm (range, 0‒1.5 mm) and 0.6 ± 0.6 mm (range, 0‒1.3 mm), respectively (Table 2). Bony union was obtained in all patients at a mean of 9.6 ± 2.1 weeks (range, 8‒12 weeks). There were two complications: In one patient, loosening of one of the two screws occurred, but the other one was maintained and union was achieved, and only this patient underwent implant removal. Two patient had adhesive capsulitis of the shoulder joint, and was cured by passive motion rehabilitation lasting 2 months. At the last follow-up after surgery, the mean constant score was 84.7 ± 11.1 points (range: 62‒98) and the mean pain visual analog scale score was 0.9 ± 0.9 (range: 0‒3). The mean active range of forward elevation, external rotation and abduction were 156.0 ± 18.9 degrees (range: 120‒180), 59.0 ± 15.9 degrees (range: 40‒80) and 153.0 ± 20.0 degrees (range: 120‒180), respectively (Table 3).

**Table 2:** Radiological results.

Patient number	Fracture type (Ideberg)	Time to bony union (weeks)	Step off (mm)		Displacement (mm)	
			Preop	Postop	Preop	Postop
1	II	12	7.86	0.24	11.72	0.54
2	II	8	2.35	1.22	4.42	1.06
3	II	8	8.78	0	15.21	0.53
4	II	8	1.47	1.7	5.61	0.67
5	II	8	3.19	0	5.17	0.53
6	II	8	3.93	0.88	5.53	1.47
7	II	12	6.71	0	6.54	0
8	II	12	2.21	1.26	14.3	1.33
9	Ib	12	7.88	0.53	12.08	1.12
10	II	8	2.21	0.53	9.94	0.53
Mean ± SD		11.1 ± 1.6	4.7 ± 2.8	0.6 ± 0.6	9.1 ± 4.1	0.8 ± 0.5

*SD* standard deviation.

**Table 3:** Clinical results at final follow up.

Patient number	Pain VAS score	Constant score	Active range of motion		
			Forward elevation	External rotation	Abduction
1	2	87	160	60	150
2	0	92	170	70	170
3	0	85	150	60	150
4	1	69	120	40	120
5	0	92	170	80	170
6	1	92	170	70	160
7	1	85	160	50	160
8	1	98	180	80	180
9	0	85	150	40	150
10	3	62	130	40	120
Mean ± SD	0.9 ± 0.9	84.7 ± 11.1	156.0 ± 18.9	59.0 ± 15.9	153.0 ± 20.0

*VAS* visual analogue scale, *SD* standard deviation.

All data generated or analysed during this study are included in this published article and its Supplementary information files.

## Discussion

In this study, the approach used for surgical repair of glenoid fractures is convenient to use with fluoroscopy and allows reduction and fixation without damaging the posterior deltoid muscle. Clinical and radiologic follow-up of our patients showed good results.

Conservative treatment has generally been recommended for scapular fractures; however, surgical treatment is recommended for displaced intra-articular glenoid fractures to reduce the risk of posttraumatic osteoarthritis or shoulder instability^6^. The indications for surgical management include an articular gap or step-off of between 3 and 10 mm, 20% and 30% involvement of the articular surface, and instability of the glenohumeral joint^6–10^. Our indication for treatment was an articular fracture gap or step-off of ≥ 3 mm.

In previous studies, most surgical treatments used Judet’s approach, and good results were reported^6,9,11–14^. However, this is an extensive approach that causes marked damage to the soft tissue and deltoid muscle. Moreover, in the lateral decubitus position, because fluoroscopy cannot be easily implemented, this approach is challenging for surgeons who lack experience. Brodsky introduced a simple posterior approach technique, but did not report clinical outcomes or clinical images^2^. It has been reported that soft tissue damage can be minimized by performing arthroscopic treatment^15–20^. This is mainly performed for Ideberg type Ia or III factures, and is difficult to apply for Ideberg type Ib or II. For Ideberg type II fractures, ideal fixation (perpendicular to the fracture line) requires screw insertion at 6 o’clock (Fig. 4), but arthroscopic fixation is difficult, because of the risk of axillary nerve injury.

In this study, a simple and effective approach was introduced, and correct reduction was achieved for posterior open reduction, without deltoid damage or extensive soft tissue dissection as is required with Judet’s approach. Depending on the angle of the fracture line, one or two screws were inserted from 6 to 8 o’clock, and sufficient fixation force was obtained. A 4.0-mm partial-thread cannulated compression screw was used to fix it through the far cortex, because cancellous bone fixation was not sufficient to obtain strong fixation of the glenoid fracture. Cortical screws could be fixed in the same way, but it was difficult to ascertain the appropriate direction of the screw before insertion. In the case of patients with well-developed muscles or severe obesity, access through the same incision could be difficult. In this study, there were two patients whose body mass index (BMI) was over 30, but surgery was possible through this approach by extending the skin incision.

When inserting a screw or pin at the 6 o’clock position of the glenoid, there was concern about damage to the axillary nerve. As mentioned in the previous surgical method, safe access was possible by retracting the long head of the triceps muscle toward the lateral side along with the axillary nerve. In some fractures, when screws must be inserted close to the articular surface, the long head of the triceps muscle could be partially detached, but complete resection should be avoided.

In this case series, patients with scapular body fractures requiring surgery were excluded. Plate fixation was possible for scapular neck fractures through this approach, but Judet’s approach was necessary for patients requiring surgery for the scapular body fractures. In one patient, the anterior cortical bone of the scapula was penetrated more than 5 mm, and the tip of the screw was placed inside the subscapularis muscle. However, this patient did not complain of discomfort, so it was not removed. One screw loosening occurred 6 months after the surgery. Although no discomfort was reported, implant removal was performed after confirmation of union by CT scan. There was one case of preoperative axillary nerve palsy had recovered by 3 months after injury.

There are two strengths to our study. First, to our knowledge, no previous study has evaluated the outcomes after ORIF of glenoid fossa fractures by this deltoid-sparing approach to access the joint. Second, the state of joint reduction was evaluated by CT scan after surgery: most previous studies used only simple radiography for postoperative assessment. However, simple radiography alone does not provide an accurate assessment of the gap and step-off of the glenoid fossa.

Our study has several limitations. The number of cases enrolled in our study was small, due to the rarity of this fracture. Secondly, the follow-up time was too short to allow further analysis of the risk factors for posttraumatic arthritis; however, it was not considered to be insufficient to evaluate the maintenances of fixation, bony union, and clinical outcomes. Another concern was the radiation exposure from the postoperative CT scan. Since the scan proved that the reduction and fixation with the posterior deltoid-sparing approach is excellent, in future surgeries, we will perform CT scans only when the reduction is not satisfactory.

## Conclusion

The posterior deltoid-sparing approach is an effective and easy method for the treatment of inferior or posterior glenoid fossa fractures (Ideberg type II or Ib).

### Ethical approval

Chungbuk National University Hospital IRB File No 2020-11-009-002.

### Supplementary Information

Supplementary Information.
